# Selective Deletion of PTEN in Dopamine Neurons Leads to Trophic Effects and Adaptation of Striatal Medium Spiny Projecting Neurons

**DOI:** 10.1371/journal.pone.0007027

**Published:** 2009-09-11

**Authors:** Oscar Diaz-Ruiz, Agustin Zapata, Lufei Shan, YaJun Zhang, Andreas C. Tomac, Nasir Malik, Fidel de la Cruz, Cristina M. Bäckman

**Affiliations:** 1 Cellular Neurobiology Branch, National Institute on Drug Abuse, National Institutes of Health, Baltimore, Maryland, United States of America; 2 Behavioral Neuroscience Branch, National Institute on Drug Abuse, National Institutes of Health, Baltimore, Maryland, United States of America; 3 Escuela Nacional de Ciencias Biológicas, Instituto Politécnico Nacional, Mexico City, Mexico; Columbia University, United States of America

## Abstract

The widespread distribution of the tumor suppressor PTEN in the nervous system suggests a role in a broad range of brain functions. PTEN negatively regulates the signaling pathways initiated by protein kinase B (Akt) thereby regulating signals for growth, proliferation and cell survival. *Pten* deletion in the mouse brain has revealed its role in controlling cell size and number. In this study, we used Cre-*loxP* technology to specifically inactivate *Pten* in dopamine (DA) neurons (*Pten* KO mice). The resulting mutant mice showed neuronal hypertrophy, and an increased number of dopaminergic neurons and fibers in the ventral mesencephalon. Interestingly, quantitative microdialysis studies in *Pten* KO mice revealed no alterations in basal DA extracellular levels or evoked DA release in the dorsal striatum, despite a significant increase in total DA tissue levels. Striatal dopamine receptor D1 (DRD1) and prodynorphin (PDyn) mRNA levels were significantly elevated in KO animals, suggesting an enhancement in neuronal activity associated with the striatonigral projection pathway, while dopamine receptor D2 (DRD2) and preproenkephalin (PPE) mRNA levels remained unchanged. In addition, PTEN inactivation protected DA neurons and significantly enhanced DA-dependent behavioral functions in KO mice after a progressive 6OHDA lesion. These results provide further evidence about the role of PTEN in the brain and suggest that manipulation of the PTEN/Akt signaling pathway during development may alter the basal state of dopaminergic neurotransmission and could provide a therapeutic strategy for the treatment of Parkinson's disease, and other neurodegenerative disorders.

## Introduction

*Pten* (for ‘phosphatase and tensin homologue deleted on chromosome ten’) is a tumor suppressor gene mutated in many human cancers, including glioblastomas, a highly malignant glial tumor in the CNS [1]. Individuals with germline *Pten* mutations are prone to tumors and may display brain disorders, including macrocephaly, seizures and mental retardation [2].

The tumor suppressive property of PTEN is dependent on its lipid phosphatase activity, which restrains the activation of the Akt (also called protein kinase B) signaling pathway. Upon activation, Akt phosphorylates a diverse spectrum of substrates known to regulate cellular functions related to cell cycle progression, cell growth and proliferation, cell death/survival and cell differentiation [3].

PTEN is widely distributed in the brain and is preferentially expressed in neurons, where it localizes to both the nucleus and cytoplasm [4]–[7]. The role of PTEN in the brain has been largely focused on the pathogenesis of glioblastoma; however, progress has recently been made in understanding the broader role of PTEN in neural circuits. A number of *in vitro* studies indicates a role for PTEN signaling in neuronal size, dendritic and axonal branching and neuronal polarization [8], [9]. To analyze the role of PTEN *in vivo* and in specific neuronal populations mutant mice have been generated to induce the loss of the phosphatase in a tissue-specific manner by using the *Cre-LoxP* technology. Conditional loss of *Pten* in the brain can have differing consequences depending on the cell type or state of differentiation. For example, the use of a Nestin promoter to induce *Pten* deletion in neural stem cells and glial progenitors resulted in neonatal death, as mice showed enlarged and histoarchitecturally abnormal brains [10]. *Pten* deletion in discrete mature neuronal populations in the cerebral cortex and hippocampus resulted in macrocephaly due to neuronal hypertrophy. In addition abnormalities in axonal growth and synapse number resulted in abnormal social behavior and inappropriate responses to sensory stimuli [11].

Recent studies have emphasized the association of PTEN with different parameters of the dopaminergic system in the ventral midbrain. For example, the continuous activation of the downstream PTEN pathway, Akt, in mesostriatal adult dopamine neurons by unilateral adenoviral injections in the substantia nigra compacta confers almost complete protection against apoptotic cell death in a dopamine toxin specific model [12]. In addition, a recent study has shown Akt signaling in the mesolimbic dopamine system may also regulate functions intrinsic to dopamine neurons, such as cellular and behavioral responses to stressful stimuli [13]. Besides the inhibitory control on the activation of the Akt pathway, PTEN interacts with molecular substrates directly involved in neurotransmission such as glutamate and serotonin receptors [13], [14]. Normally the activity of mesolimbic dopamine cells is under the tonic inhibitory control of the phosphorylated serotonin receptor 2c (5-HT2c). PTEN deletion in DA cells favors the phospholylated state of the 5-HT2c receptor, inhibits dopaminergic transmission and abolishes the rewarding effects of nicotine and tetrahydrocannabinol [13]. These studies suggest PTEN function in dopamine neurons is not limited to the classical deregulation of the Akt pathway, and PTEN may also have an effect on distinct functions related to dopamine-mediated cognition. To further examine the correlation between PTEN and the dopaminergic system, we developed a mouse transgenic model to inactivate PTEN signaling specifically in DA neurons. We now report that PTEN ablation during development results in profound morphological, molecular and neurochemical changes in DA neuron maturation that translate into permanent adaptations of the dopaminergic system, including significant changes in postsynaptic brain areas. Additionally, and in agreement with previous studies [12], we show that PTEN deletion in dopamine cells provides significant protection against neurotoxic insults. Such adaptations will be documented in detail in this report. Future studies will be aimed at correlating the molecular adaptations mediated by PTEN deletion with behavioral aspects of dopamine neurotransmission.

## Methods

### Mice

*Pten^loxP^* mice were obtained from Jackson laboratories [14]. Mice were obtained in a background comprised of a mix of c57bl/6, 129S4/SvJae. All animals used in this study were analyzed between 3–4 months of age. Animal protocols used in this study were approved by the Animal Care Committee at the National Institutes of Health. For the conditional inactivation of *Pten* in dopaminergic neurons of the ventral midbrain, we developed a dopamine transporter (DAT) promoter-driven Cre transgenic mouse line *Slc6a3* ^Cre^ [15]. Cre recombinase binds to *loxP* target sequences and either deletes or inverts the intervening DNA depending on the individual orientation of the *loxP* sites [16]. In our model, Cre recombinase expression is driven by the DAT promoter and mediates deletion of exon 5 of the *pten* gene approximately at embryonic day 15 [15]. DAT is a molecular substrate specific to dopamine neurons and is highly expressed in dopamine neurons of the ventral midbrain, while lesser expression levels are present in the olfactory bulb and hypothalamic areas. To minimize interference with DAT function by preservation of both DAT alleles, Cre recombinase expression was driven from the 3′-untranslated region (3′UTR) of the endogenous DAT gene by means of an internal ribosomal entry sequence (IRES). The *Pten^loxP^* and *Slc6a3* ^Cre^ lines were mixed to obtain regional knockout (*Pten ^loxP/loxP^* ^/Cre/wt^) and control mice (*Pten* ^wt/wt/Cre/wt^). Animals were genotyped using *Pten* and *Slac6a3*-Cre primers described elsewhere [14], [15].

To confirm if *Pten* deletion was specific to DAT expressing regions, primers specific to the recombination (delta5) were developed. Genomic DNAs were prepared from the olfactory bulb, motor cortex, striatum and SN/VTA. For ventral midbrain dissections, coronal cuts were made at the anterior and posterior boundaries of the mammillary nucleus. Dissection of the ventral midbrain region containing the SN/VTA was facilitated by removing the mammillary nucleus on the ventral surface and overlying cortex on the dorsal surface. A block containing the SN/VTA was then dissected. To further ensure specificity of Cre mediated recombination lung, heart, kidney and liver tissue was dissected and DNA extracted for PCR analysis.

### Immunohistochemistry

Adult mice were deeply anesthetized with an intraperitoneal (i.p.) injection of chloral hydrate (30 mg/kg) and then perfused transcardially with saline solution followed by 4% paraformaldehyde (PFA). Brains were quickly removed from skull and post-fixed in 4% PFA for 4 hrs. After fixation, brains were rinsed in 0.1 M phosphate buffer (PB) and cryoprotected in 18% sucrose solution, overnight at 4°C.

Striatum and midbrain regions were cut in coronal sections at 40 um in four series using a Leica CM3050S cryostat (Leica Microsystems, Bannockburn, IL). Sections were processed for Tyrosine Hydroxylase (TH) immunohistochemistry or for Pten or phosho-AKT immunofluoresce. For TH immunohistochemical studies, sections were rinsed with PB 3×10 min and then permeabilized and blocked with 0.25% Triton-X-100 and 4% bovine serum albumin (BSA) in 0.1 M PB. Sections were incubated overnight at 4°C with a rabbit polyclonal antibody against TH (1∶1000, Chemicon, Temecula, CA). Sections were then rinsed 3×10 min in PB, and incubated for 1 hr with a biotinylated anti-rabbit antibody (1∶200, Vector Labs, Burlingame, CA). Sections were rinsed with PB 3×10 min and incubated with avidin-biotinylated horseradish peroxidase for 2 h. Sections were rinsed and the peroxidase reaction was developed with 0.05% 3,3-diaminobenzidine-4 HCl (DAB) and 0.003% hydrogen peroxide (H_2_O_2_). Sections were mounted on coated slides, coversliped and dried before analysis.

For immunofluorescence histochemistry, sections were rinsed in PB 3×10 min, and then incubated with 0.25% Triton-X-100 and 4% BSA in 0.1 M PB. Sections were incubated with a mouse monoclonal anti PTEN (Cell Signaling, Danvers, MA) or with anti p-AKT rabbit monoclonal antibody (1∶100, Cell Signaling, Danvers, MA) overnight at 4°C. Sections were rinsed with PB 3×10 min and incubated for 2 hrs at room temperature with one of the following secondary fluorescent antibodies: anti mouse or anti rabbit (Invitrogen, Carlsbad, CA), rinsed with PB 3×10 min, mounted on coated slides and coversliped. Sections were analyzed in a Leica DMLA microscope (Leica Microsystems, Bannockburn, IL) with fluorescent light.

### Stereologic Analyses

Unbiased stereological counts of TH-positive (TH+) neurons within the substantia nigra pars compacta (SNc) and ventral tegmental area (VTA) as well as fiber length in the substantia nigra pars reticulata (SNr) were performed using stereological principles [17] and analyzed with StereoInvestigator software (Microbrightfield, Williston, VT). The optical fractionator probe [18] was used to generate an estimate of neuronal TH+ number and area, nucleator probe [19] to estimate the size of TH+ neurons in SNc and VTA and space balls probe [20] to obtain an estimate of the total length of fibers in the SNr. SNc, VTA and SNr were outlined under a low magnification objective (5x) following landmarks from the Franklin and Paxinos mouse atlas [21] and all the stereologic analysis were performed under the 40x objective of a Leica DM5000B microscope (Leica Microsystems, Bannockburn, IL).

The total number of TH+ neurons, neuronal size and fiber density, was estimated for adult naïve control and *Pten* KO mice. For each tissue section analyzed, section thickness was assessed in each sampling site and guard zones of 2.5 µm were used at the top and bottom of each section. Systematic random sampling design was performed and generated with the following stereologic parameters: grid size: 131 µm, counting frame: 123 µm and dissector height of 25 µm. For space balls a 20 µm radius was used to intersect fibers that crossed the circle. The criterion for a TH+ fiber was a stained and in focus fiber at the crossing point in the circle. Our criterion for counting and measuring the area of an individual TH+ neuron was the presence of its nucleus either within the counting frame, or touching the right or top frame lines (green), but not touching the left or bottom lines (red). The area of TH+ neurons in the SNc and VTA was estimated using the nucleator probe. Every time a neuron was counted with the optical fractionator probe, a set of four rays are extended from the middle of the cell and radiate with a random orientation in four orthogonal directions towards the edge of the neuron. The point at which each ray intersected the boundary of the neuron are used to define the area. Coefficients of error were calculated and values <0.10 were accepted.

Stereologic estimations were also performed with the same parameters in the SNc of control and *Pten* KO mice thirty days after receiving a unilateral 6OHDA lesion in the striatum. Results were presented as a percentage of surviving neurons on the contralateral side.

### Optical Density

To determine fiber density in the striatum, the mean optical density (O.D.) was measured in TH positive stained sections from naïve and 6OHDA-lesioned mice (thirty days after 6-OHDA unilateral lesion) in control and *Pten* KO mice. O.D. is a sensitive and reliable tool to measure levels of fiber innervation and to detect changes by experimental manipulations [22]. Images were captured with a Hitachi CCD HV-C20A camera and transformed to grey scale images of 8 bits. The O.D. quantification was perform using ImageJ software [23]. The O.D. measures were determined in each mouse at three coronal levels corresponding to the frontal striatum +1.5 mm, medial striatum +0.14 mm. and caudal striatum −0.50 mm, relative to bregma [21]. Nonspecific background was determined by readings made from the anterior commissure or corpus callosum. For naïve animals, the O.D value corresponds to the mean of both striatae minus the respective background level. For 6OHDA-treated animals, O.D. values were measured on the denervated and non denervated striatum in control and KO mice and the O.D. minus the background was expressed as a percentage relative to the contralateral side. In addition, striatal total area was determined with the same software and results expressed as square pixels.

### Behavioral Test: Locomotor activity induced by exposure to a novel environment

All mice used were adult and kept on a 12:12 light – dark cycle (lights on 06:00 am) with food and water *ad libitum*. Behavioral tests were performed between 10:00–14:00 hrs. Locomotor activity assessed in the open field, is considered a well organized behavior in rodents determined by several factors, including anxiety, arousal, risk assessment, and exploration, and is an established tool for behavioral phenotyping in mice [24]. Control and *Pten* KO mice were brought to the testing room 30 minutes before the procedure began for acclimation. After this period, each mouse was gently placed inside a clean Plexiglas chamber (42×42×30 cm; Accuscan Instruments, Columbus, OH) for 30 minutes to assess locomotor activity in a new environment. Locomotor activity was measured in each cage by 16 horizontal and 16 vertical sensors (infrared beams) spaced 2.5 cm apart. Vertical sensors were located 7 cm above the floor chamber. Data was stored every 5 min and presented as cumulative values after 30 minutes.

### Real time PCR

Striatal tissue was dissected and immediately processed for RNA isolation and DNAse I treatment using the RNAqueous-Micro Ambion kit (Applied Biosystems- Ambion, Austin, TX), following the manufacturers instructions. For cDNA synthesis total RNA was mixed following the manufacturers instructions for the Superscript III reverse transcriptase kit (Invitrogen, Carlsbad, CA). Real-time polymerase chain reaction was performed as duplicate determinations with specific Taqman probes from the mouse Probe Library (Exiqon A/S, Vedbaek, Denmark) designed for primers to mouse DRD1 (F: 5′- gag cgt agt ctc cca gat cg-3′ and B: 5′- tgg tca atc tca gtc act ttt ca- 3′), DRD2 (F: 5′-ctc ttt gga ctc aac aac aca ga- 3′ and B: 5′ -aag ggc acg tag aac gag ac- 3′), preproenkephalin (F: 5′ -aag ggc acg tag aac gag ac- 3′ and B: 5′ -aag ggc acg tag aac gag ac- 3′), prodynorphin (F: 5′-aag ggc acg tag aac gag ac- 3′ and B: 5′-cgc cat tct gac tca ctt gtt- 3′), and BDNF (F: 5′ -gca tct gtt ggg gag aca ag- 3′ and B: 5′-tgg tca tca ctc ttc tca cct g- 3′). Hydroxymethylbilane synthase (Hmbs, F: 5′-tcc ctg aag gat gtg cct ac-3′ and B: 5′- aca agg gtt ttc ccg ttt g- 3′) and aminolevulinate synthase (ALAS, F:5′- cca tca att acc caa cag tgc-3′ and B: 5′-gtg acc agc agc ttc tcc a-3′) were used as housekeeping genes to normalize quantification data. The reproducibility of results was determined by inspection of duplicate samples. After an initial incubation step for 10 min at 95°C, qRT-PCR was carried out using 40 cycles (95°C for 15 seconds, 60°C for 60 seconds). The standard curve method was used to compare mRNA expression levels between KO and control animals. Normalization to both endogenous control genes led to similar results.

### *In Vivo* Microdialysis

*Pten* deficient mice and controls were anesthetized and implanted unilaterally with a microdialysis guide cannula (CMA/11, CMA microdialysis) in the dorsal striatum (AP: +0.4, L: -2.1, V: -2.2 mm from bregma) using standard stereotaxic techniques, and were allowed to recover for 5 days before the microdialysis experiments [25]. Fifteen hours before the start of experiments, a microdialysis probe (CMA/11, 2 mm membrane length, CMA microdialysis, North Chelmsford, MA) was connected to the dialysis system and flushed with artificial cerebrospinal fluid (aCSF: 145 mM NaCl, 2.8 mM KCl, 1.2 mM CaCl_2_, 1.2 mM MgCl_2_, 0.25 mM ascorbic acid, and 5.4 mM D-glucose, pH 7.2 adjusted with NaOH 0.5 M). The mouse was gently restrained and the probe was slowly inserted into the guide cannula. The dialysis system consisted of FEP tubing (CMA microdialysis) that connected the probe to a 1 ml gastight syringe (Hamilton Co., Reno, NV) mounted on a microdialysis pump (CMA/102) through a quartz-lined, low-resistance swivel (375/D/22QM, Instech, Plymouth Meeting, PA). After probe insertion, the mouse was placed in the dialysis chamber with food and water freely available, and the probe perfused overnight with aCSF at a flow rate of 0.3 µl/min. The next morning, the perfusion syringes were loaded with fresh aCSF and probes were allowed to equilibrate for an additional 1 h at a flow rate of 0.6 µl/min before the start of experiments.

For no net flux experiments, five different concentrations of DA (0, 5, 10, 20, and 40 nM) in aCSF were perfused in random order through the dialysis probe. Each DA concentration was perfused for 30 min, and then 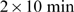 samples were collected. Following completion of the no net flux experiments, normal aCSF was again perfused through the probe for 30 min, allowing for a period of equilibration. Consecutive 15 min samples were then collected. Three baseline samples were then collected followed by changing the perfusion buffer to aCSF containing 60 mM KCl (NaCl concentration was reduced accordingly to maintain osmolarity) and four samples were obtained. The perfusion buffer was switched back to normal aCSF and three additional samples were collected. After the experiments, mice were killed by pentobarbital overdose and their brains were removed. The midbrain and left dorsal striatum were dissected out, frozen on dry ice and stored at −80 for tissue *DA* content determination. The right forebrain was frozen on dry ice, and 20 µm sections were obtained on a cryostat for the histological verification of probe location. No net flux data were analyzed as described [25]. The net flux of DA through the probe (DAin–DAout) was calculated and plotted against the concentration of DA perfused (DAin). The following parameters were calculated from the resulting linear function. The *y* axis intercept, corresponding to zero DA perfused through the probe is the dialysate DA concentration in a conventional microdialysis experiment. The *x* axis intercept corresponds to the point of equilibrium where there is no net flux of DA through the probe and provides an unbiased estimate of extracellular DA concentration. The slope of the regression line corresponds to the extraction fraction (Ed) which has been shown to be an indirect measure of DA uptake [25], [26]. The resulting regression lines were compared by Fisher's test using GraphPad Prism software. In the conventional microdialysis experiments investigating the effects of 60 mM KCl, the average of the three baseline samples was calculated, and all the DA concentrations were expressed as % of that baseline. Differences between control and *Pten* KO animals in the appropriate variables were assessed by comparing both groups using a Student's *t*-test.

DA was determined by HPLC coupled to electrochemical detection. The chromatographic system consisted of a CMA/200 refrigerated microinjector (CMA microdialysis, North Chelmsford, MA), a BAS PM-80 pump (BAS, West Lafayette, IN) and a BAS LC-4C amperometric detector. The mobile phase (0.15 M sodium phosphate, 2.24 mM sodium octanesulfonic acid, 0.94 mM EDTA, adjusted to pH 5.0 and containing 11% methanol (vol/vol) was filtered through a 0.22-µm nylon filter, degassed by a BAS online degasser, and pumped through the system at a flow rate of 0.6 ml/min. DA was separated in a BAS C18 column (100 mm×2.0 mm×3 µm) and detected on a glassy carbon working electrode at an oxidation potential of +700 mV vs. Ag/AgCl. Dialysate DA levels were quantified by external standard curve calibration, using peak heights for quantification. All the reagents used for the mobile phase were analytical grade. Under these conditions, retention time for DA was 3 min, and the limit of detection was 0.25 nM.

### Tissue Levels of DA and metabolites

The midbrain and the left dorsal striatum were dissected after the microdialysis experiments and stored at −80C for the determination of monoamine content. Tissue samples were homogenized by ultrasonication in 20 volumes of ice cold 0.05N HClO_4_ containing 0.5 µM of DHBA as the internal standard. Homogenates were centrifuged at 22000 g for 10 min at 4C. Aliquots from the supernatant were analyzed for monoamine content by HPLC coupled to electrochemical detection. The chromatographic system was similar to the one described for microdialysis experiments except for the column (100×1 mm C18,5 um particle size, BAS) and the mobile phase (35.2 mM sodium phosphate, 26.4 mM citric acid, 1.66 mM sodium octanesulfonic acid and 0.1 mM EDTA adjusted to pH 4.2; containing 6% methanol and 0.4% tetrahydrofurane, vol/vol). The concentrations of monoamines were calculated against an external standard curve, normalized by the internal standard and corrected by the protein concentration in the homogenate.

### 6-Hydroxydopamine (6OHDA) Lesions

Mice were anesthetized with Avertin, and placed in a stereotaxic frame. 6OHDA (5.0 µg/µl in 0.9% NaCl/0.02% ascorbate) was injected using a microliter syringe at a rate of 0.5 µl/min for a total dose of 15.0 µg/3 µl. Injection was performed into the striatum at coordinates AP: +0.09 cm; ML: ±0.22 cm; DV: −0.25 cm relative to bregma. After 2 min, the needle was withdrawn slowly. For amphetamine-induced rotation, mice were injected with (+) methamphetamine HCl (2.5 mg/kg i.p. injection) and placed in a rotometer (Accuscan, Columbus, OH) for 120 min. Baseline amphetamine rotation tests were performed 7 days before 6OHDA lesions and the results used to assign the side of the subsequent 6OHDA lesion. Mice exhibiting net clockwise turns were lesioned in the left striatum, while mice exhibiting net counter-clockwise turns were lesioned in the right striatum. Following the 6OHDA lesion, amphetamine-induced rotational behavior was tested 14 and 28 days after lesion. The number of clockwise and counter-clockwise turns was counted and expressed as the number of net rotations per 120 minutes to the lesioned (ipsilateral) hemisphere.

### Statistical analyses

Unless otherwise stated, statistical analyses between control and *Pten* KO animals were performed by using a Student's t test with values of *p<0.05 considered significant.

## Results

### *Pten* deletion is specific for cells expressing the dopamine transporter (DAT)

To establish a mouse model carrying *Pten* deletion in cells expressing DAT, we crossed *Pten loxP* mice with a *Slc6a3* ^Cre^ transgenic line. Of the offspring generated, we used *Pten^ loxP/loxP^* ^/Cre/wt^ as knockouts (KO) and *Pten* ^wt/wt/Cre/wt^ as experimental controls. *Pten* KO mice were born at the expected Mendelian ratio and were visibly indistinguishable from control mice.

**Figure 1:**
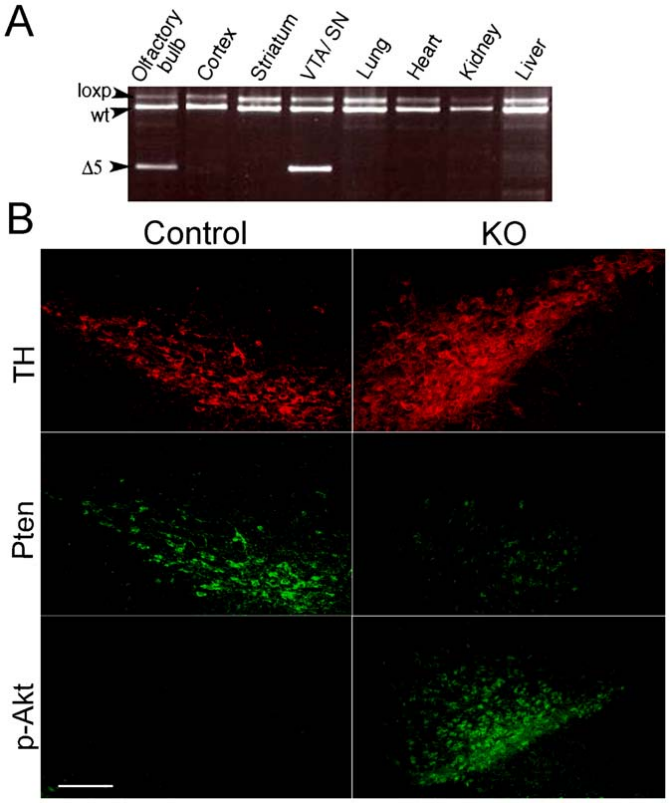
Cre recombinase deletes *Pten* exon 5 in neurons expressing the dopamine transporter. (A) PCR screen for Cre-mediated *Pten* exon5 deletion. Genomic DNAs were prepared from the indicated brain tissues from a *Pten ^loxP/loxP^* ^/Cre/wt^ adult mouse. PCR analysis show *Pten* deletion (delta5) is specific to the olfactory bulb and SN/VTA. *Pten* deletion could not be detected in other brain areas analyzed. (B) Immunofluorescent staining shows TH and PTEN staining in the ventral mesencephalon of control and *Pten* KO animals. In the mutant animals, loss of PTEN activity is associated with increased TH and membrane phospho-AKT staining. Sections correspond to Bregma -2.95 mm with reference to Franklin and Paxinos (1997). Scale bar, 250 µm.

To confirm Cre-mediated recombination in *Pten ^loxP/loxP^* ^/Cre/wt^ mice we used PCR and immunological strategies. As shown in figure 1A, *Pten* exon5 deletion was specific to the SNc, VTA and olfactory bulb in adult animals (delta5 band), with no detectable leakage in other brain areas [15]. In addition, the extent of *Pten* inactivation in KO mice was examined by immunocytochemistry using antibodies against TH, PTEN and phospho-AKT. In KO animals the loss of PTEN immunoreactivity in the ventral mesencephalon was associated with increased TH and phospho-AKT staining (Figure 1B).

As PTEN is a tumor suppressor gene and deregulation of this pathway has been associated with the formation of multiple tumor types, the animals used for morphological evaluations in this study were examined for the existence of brain tumors. Sections through the entire brain did not show evidence of neoplastic transformation in any of the brains analyzed (data not shown).

### *Pten* deletion leads to neuronal hypertrophy and increased number of TH positive neurons and dendrites in the ventral midbrain

DA midbrain neurons are first generated near the midbrain-hindbrain junction and migrate radially to their final position in the ventral midbrain [27], [28]. Tyrosine hydroxylase (TH), the rate-limiting enzyme in the biosynthetic pathway of cathecholamines, can be first detected in the mouse at embryonic day (E) 11.5, suggesting initiation of DA differentiation [27]. In contrast, DAT gene transcripts are not detected in the mouse ventral mesencephalon until DA axons reach the target at about E15, suggesting that during ontogeny, DA synthesis, and high-affinity uptake develop asynchronously and in a non-correlated fashion [27]. In DAT-Cre conditional transgenic mice, Cre recombinase activity is induced shortly after DAT induction. In a previous report we first detected DAT-Cre mediated beta galactosidase expression in a conditional Rosa26LacZ reporter mice at embryonic day 17 [15], suggesting excision of *Pten* exon5 at around this developmental point. Stereological cell counts of TH positive profiles in the substantia nigra and VTA were significantly elevated already at postnatal day two (data not presented), indicating an early expression of anatomical changes mediated by *Pten* ablation.

**Figure 2:**
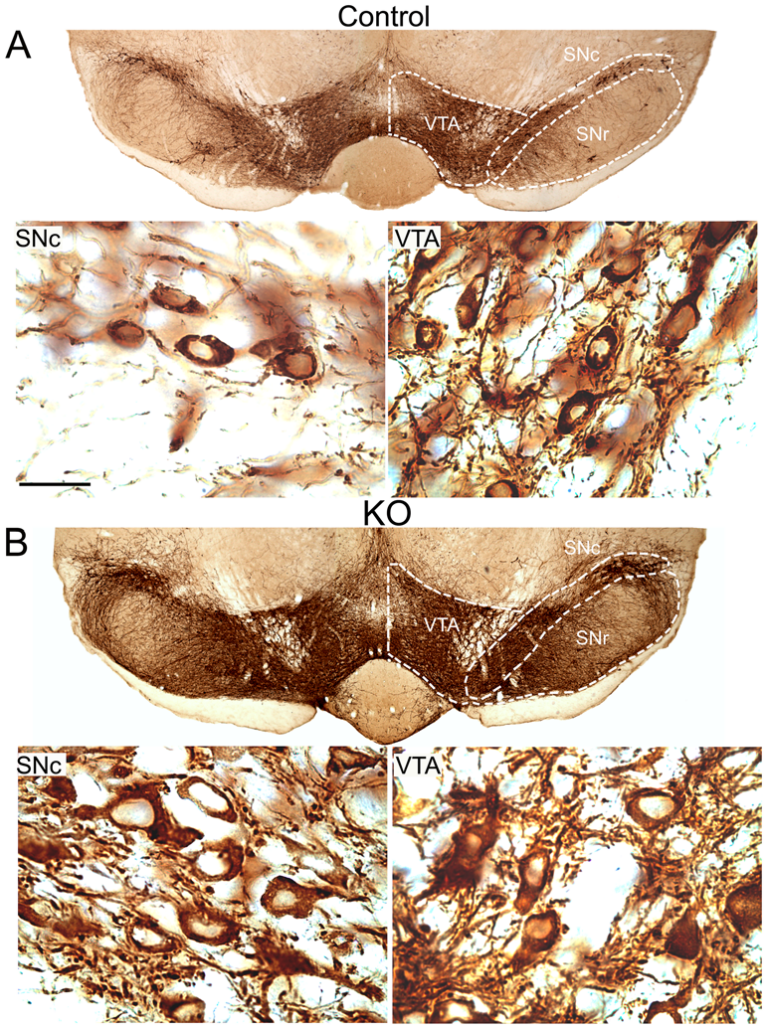
*Pten* deletion alters DA neurons in the ventral midbrain. Sections processed for TH immunostaining show a significant increase in the number of TH immunopositive profiles in the substantia nigra compacta (SNc) and ventral tegmental area (VTA) of adult *Pten* KO animals when compared to controls (A and B). The total area covered by TH positive staining was significantly increased in *Pten* KO animals when compared to controls. Increase in the size of the mesolimbic and nigrostriatal projecting structures are due not only to an increase in the number of DA neurons, but also to an increase in neuronal size and in dendritic outgrowth (see inserts in A and B). Sections correspond to Bregma -3.16 mm with reference to Franklin and Paxinos (1997). Scale bar corresponding to high magnification, 250 µm, and low magnification, 25 µm.

**Figure 3:**
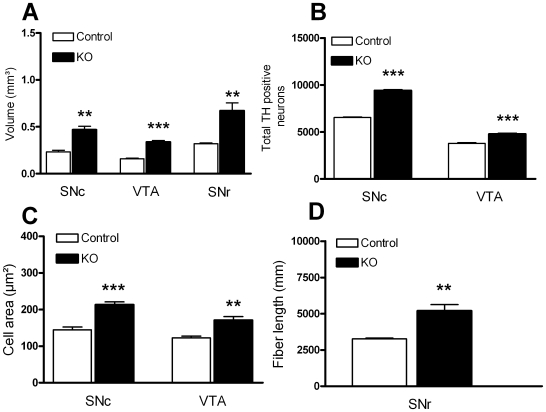
The lack of PTEN in DA neurons results in a significant enlargement of the ventral midbrain area, attributable to an increase in the total number of DA neurons that are larger in size, and display more dendritic extensions. (A) The volume of the substantia nigra compacta (SNc), ventral tegmental area (VTA) and substantia nigra reticulata (SNr) was significantly larger in *Pten* KO animals (n = 4), as compared to controls (n = 4). The volume of all measured areas was at least 40% larger in KO animals, with the largest increment in size seen in the SNr. (B) The increase in volume of the SNc and VTA from *Pten* KO animals was accompanied by a significant increase in the number of TH positive neurons (B) and cell size (C) in both regions. (B) The mean number of TH positive profiles in *Pten* KO animals was 9401±105.1 in the SNc, and 4775±106.3 in the VTA, a 43% and 26% increase, respectively, when compared to controls. (C) The area of TH positive neurons was 47% and 39% larger in the SNc and VTA of *Pten* KO animals as compared to controls. (D) The increase in the total volume covered by the SNr was attributable to an increase in the number and caliber of TH-positive fibers present in *Pten* KO animals in parallel with an increase in the number and size of TH positive cells in the SNc and VTA. All data are mean±SEM. *p<0.05, Student's t-test.

A common phenotype associated with the loss of PTEN in neuronal populations is an enlargement of neuronal soma size, increased cell proliferation, inhibition of apoptosis, and the expression of thickened axonal and dendritic processes. We performed detailed morphological analyses of brain structures affected by the mutation in adult animals. PTEN ablation in DA neurons (n = 4) induced a significant increase in SNc and VTA total volume (Fig. 2A and B; Fig. 3A) as compared to controls (n = 4). At a cellular level, this increased regional volume was mainly attributed to a significant increase in the number of neurons (Fig. 3B), as well as an increase in neuronal soma size (Fig. 2A and 2B insets and 3C). Fiber density measurements also revealed a significant increase in the number of dendritic processes in the substantia nigra pars reticulata (SNr, Fig. 3D). In many cases, dendritic processes were so close together and thickened in the SNr that at low magnification may appear as cell bodies.

**Figure 4:**
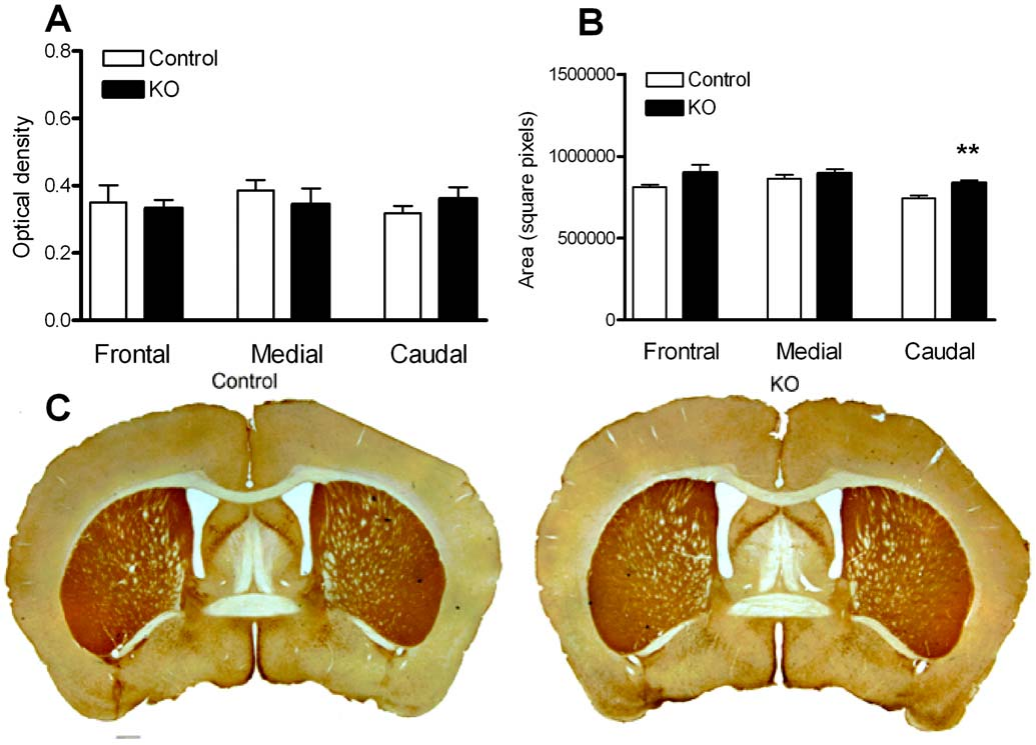
Pten KO animals do not show differences in striatal TH fiber density measurements. (A, C) Optical density values did not reveal significant differences in striatal TH staining intensity between control (n = 4) and *Pten* KO animals (n = 4). (B) There were no significant differences in area measurements corresponding to rostral and medial striatum. A slight but significant increase in area was observed in the caudal striatal region of *Pten* KO animals as compared to controls. Scale bar, 500 µm. All data are mean±SEM. *p<0.05, Student's t-test.

Interestingly, changes in neuronal size and number were not accompanied by significant differences in striatal TH staining intensity (Fig. 4A and 4C). Area measurements suggest a modest but significant enlargement of the caudal striatum in KO animals as compared to controls (Fig. 4B).

### *Pten* deletion alters the expression of postsynaptic markers in the striatum

**Figure 5:**
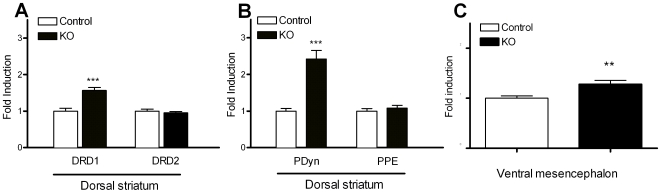
*Pten* ablation in DA cells affect the direct striatal output system by significantly increasing mRNA expression levels for DRD1 and pDyn in striatal medium spiny neurons. BDNF mRNA levels were significantly elevated in the ventral mesencephalon (A) Dopamie receptor D1 (DRD1) and D2 (DRD2) mRNA expression levels in the striatum of control (n = 9) and *Pten* KO animals (n = 13). DRD1 mRNA expression levels were significantly increased in KO animals as compared to controls. DRD2 mRNA levels were unchanged between the groups. (B) Changes in DRD1 expression levels were accompanied by an elevation in prodynorphin (pDyn) mRNA in *Pten* KO animals. Preproenkephalin (PPE) mRNA expression levels were unchanged. (C) BDNF mRNA levels were significantly increased in the ventral mesencephalon in KO animals. All data are mean±SEM. *p<0.05, Student's t-test.

Over 90% of neurons of the striatum are medium spiny projecting neurons. Distinct subpopulations of medium spiny neurons can be recognized based on their projections and expression of various markers. Direct and indirect striatal projection neurons selectively express the DRD1 and DRD2 dopamine receptor subtypes respectively [29]. Messenger RNAs encoding DRD1 are selectively localized in striatal neurons projecting to the substantia nigra (direct pathway) and co-localize with dynorphin. Conversely, mRNAs encoding DRD2 are selectively localized in neurons projecting to the lateral globus pallidus (indirect pathway) and colocalize with enkephalin. Real time quantitative PCR analyses were performed to define if *Pten* ablation altered the markers of the direct and indirect striatal pathways. Striatal mRNA levels for PPE, PDyn, and DRD1 and DRD2 receptors revealed a significant change in the expression of markers present in medium spiny neurons projecting to the substantia nigra (direct pathway). DRD1 and PDyn mRNA levels were significantly upregulated in KO animals as compared to controls (Fig. 5A and B). In contrast striatal mRNA expression levels for DRD2 and PPE did not differ between control and mutant animals (Fig. 5A and B). As striatal dynorphin positive neurons form connections with nigral DA neurons, we investigated the effects of the mutation on nigral BDNF mRNA levels. BDNF messenger levels were significantly increased in *Pten* KO animals (Fig. 5C).

### Extracellular DA Levels in Dorsal Striatum and correlation to locomotor activity

**Figure 6:**
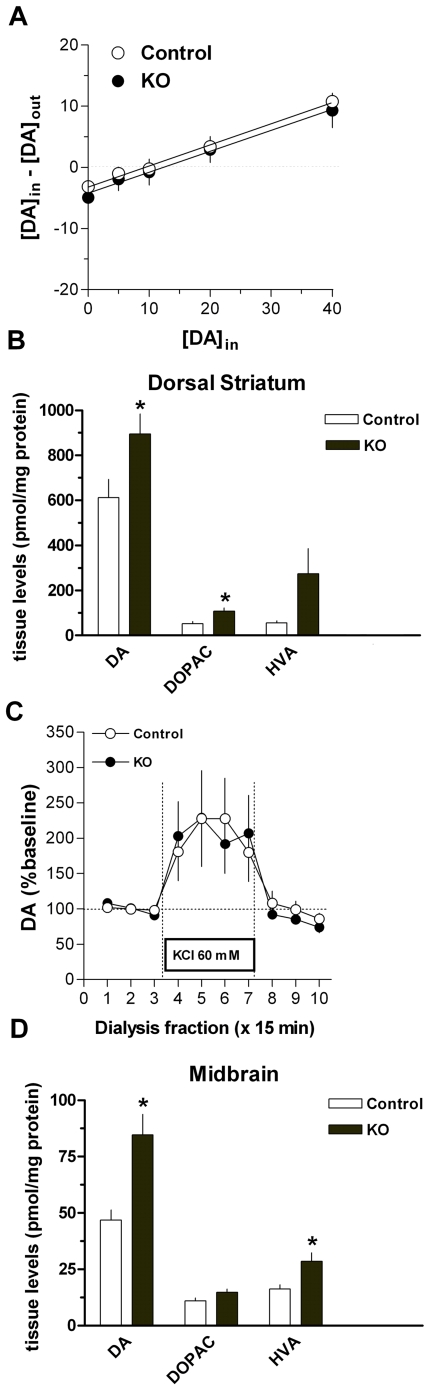
Selective *Pten* deletion in DA cells results in unaltered extracellular dopamine dynamics in spite of a significant increase in tissue catecholamine content. (A) *Pten* KO mice showed no differences in basal extracellular dopamine as estimated by the x-intercept in the no net flux analysis. DA clearance was also unaltered as indicated by no change in the extraction fraction (slope of the no net flux regression line). (C) Depolarization evoked dopamine release was unaltered in *Pten* KO animals. The total tissue content of dopamine (DA) was increased in the dorsal striatum (B) and the midbrain (D) of *Pten* KO mice. Levels for the dopamine metablites DOPAC (dihydroxyphenylacetic acid), and HVA (homovanillic acid) were significantly inceased in the striatum and midbrain respectively. 3MT (3-methoxytyramine) levels remained unchanged as compared to controls. All data are mean±SEM. *p<0.05, Student's t-test.

As previous studies have suggested levels of enkephalin and dynorphin are oppositely modulated by DA transmission, we next investigated if DA levels in the striatum were altered by *Pten* ablation in DA cells. We used the technique of *in vivo* microdialysis to investigate the consequences of genetic deletion of *Pten* in DA cells on basal extracellular DA dynamics as well as on potassium-evoked DA release. Quantitative no net flux microdialysis revealed no significant differences in basal dialysate levels (y-intercept) or in the estimated extracellular concentration (x-intercept) of DA in the dorsal striatum (Figure 6A). Moreover, the DA extraction fraction, calculated as the slope of the no net flux regression line was unchanged in KO mice, suggesting that deletion of *Pten* in DA neurons did not alter the clearance of extracellular DA by the DA transporter [26]. Conventional microdialysis revealed a marked increase in basal dialysate DA levels in response to K^+^ evoked depolarization (Fig. 6C). No significant difference between control and KO mice in KCl-evoked DA levels was observed in the dorsal striatum (fig. 6C).

**Figure 7:**
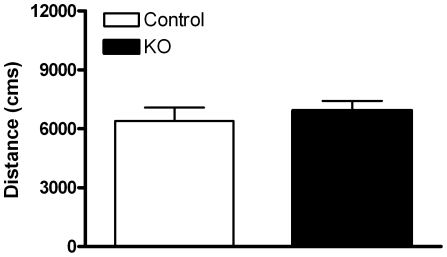
Exposure to a novel environment does not enhance locomotor activity in *Pten* KO mice. Control (n = 11) and *Pten* KO (n = 15) animals were placed in activity chambers to measure the locomotor response to a novel environment. No significant differences were found in all parameters measured during a cummulative period of 30 minutes. Total distances traveled is shown as a representative measure. All data are mean±SEM. *p<0.05, Student's t-test.

Mice lacking PTEN expression in DA cells showed an increase in catecholamine content in both the midbrain containing the DA cell bodies (fig. 6D) and in the terminal region in the dorsal striatum (fig. 6B). This increase was specific for DA and its metabolites since 5HT and 5HIAA levels were not altered (data not presented). Consistent with the dialysis data, no changes were observed in 3MT, a DA metabolite of primarily extracellular origin. In accordance with striatal DA release levels, no differences in locomotor activity were observed between control (n = 11) and *Pten* KO (n = 15) animals during exposure to a novel environment (Fig. 7).

### Pten influences neuroprotection after a neurotoxic insult

**Figure 8:**
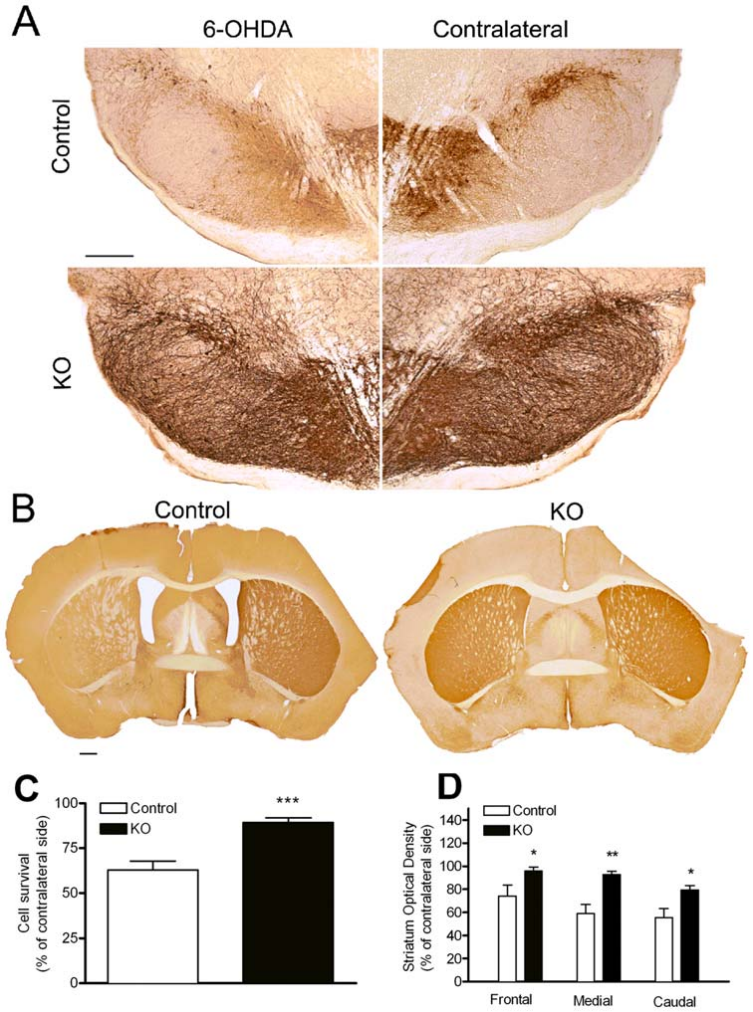
*Pten* ablation shows neuroprotective effects in the intrastriatal 6OHDA mouse model. Animals were treated with a unilateral 6OHDA injection in the striatum, and perfused 4 weeks after the injection to analyze the effect of *Pten* ablation on the magnitude of the lesion. (A) The SNc from control animals (n = 4) was markedly affected by the lesion as reflected by the significant loss of TH positive profiles in the ipsilateral side to the lesion as compared to the contralateral side. The dopaminergic neurons in *Pten* KO (n = 6) animals were largely spared after the lesion when compared to control animals. (B) The neuroprotective effects related to *Pten* ablation were observed at the level of the striatal axonal projections as well. Depletion of TH positive terminals in the striatum was reduced across the striatum in control animals, while axon terminal degeneration was much lower in *Pten* KO animals. (C) Survival rates for TH-positive neurons were quantified by stereologic counts. The number of surviving neurons (presented as percent surviving neurons when compared to the side contralateral to the lesion) was significantly increased in *Pten* KO animals (n = 6) when compared to controls (n = 4). (D) Optical density measurement in the injected striatum revealed a nearly complete preservation of TH terminals in *Pten* KO animals. TH staining density was significantly lower in control 6OHDA-treated animals with the biggest difference observed in the medial portion of the striatum, in accordance with sterotaxic coordinated used for the lesion. (A) Scale bar, 250 µm and (B) 500 µm. All data are mean±SEM. *p<0.05, Student's t-test.

A unilateral injection of 6OHDA into the striatum results in progressive loss of DA neurons in the ventral mesencephalon, and an imbalance in the levels of DA and its receptors between the two striatae [30]. We found that *Pten* deletion protects DA neurons during this partial 6OHDA lesion and prevents fiber loss in the lesioned striatum when compared to control animals. Four weeks after the lesion, control 6OHDA-treated mice showed an extensive loss of TH positive SNc neurons in the ipsilateral or lesioned nigral region (Fig. 8A and B). Stereologic cell counts in control animals showed a 63% survival rate of TH positive neurons when compared to the contralateral side. In contrast, *Pten* KO mice showed an 89% survival rate on the lesioned side as compared to the contralateral nigra (Fig. 8C). *Pten* deletion also provided significant protection against axonal loss in the lesioned striatum. Four weeks after the lesion, quantification of TH+ fiber density in the lesioned striatum showed a significant decrease in optical density levels in control animals when compared to *Pten* KO animals. Significant differences in optical density were observed in rostral, medial and caudal sections (Fig. 8D).

**Figure 9:**
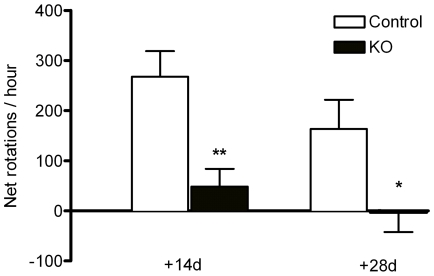
The morphological preservation of the *Pten* deficient nigrostriatal system after 6OHDA treatment correlates with functional recovery after the lesion. To determine the functionality of the nigrostriatal system after the lesion, we examined methamphetamine-induced rotations. Methamphetamine treatment increases the extracellular availability of endogenous DA in the striatum, and it causes animals with a partial DA depletion to rotate against the lesion side (ipsilateral), due to the imbalance in DA release between the striatae. Strong ipsilateral rotational behavior was observed in control animals (n = 8) after treatment with (+) methamphetamine HCl (2.5 mg/kg) at 14 and 28 days after the lesion. Ipsilateral rotational behavior was significantly reduced in *Pten* KO animals (n = 10). All data are mean±SEM. *p<0.05, Student's t-test.

The morphological preservation of the dopaminergic system observed in *Pten* KO animals was paralleled by a significant decrease in behavioral impairment developed after this partial 6OHDA striatal lesion. Unilateral 6OHDA striatal lesions result in an imbalance in the levels of DA between the two striatae. As DA stores are depleted due to the loss of DA in axon terminals, the injection of drugs that act to release DA, such as amphetamine, will induce rotational behavior towards the denervated striatum. We examined the effects of *Pten* deletion on amphetamine-induced rotational asymmetry at different time points after 6OHDA lesioning. Amphetamine-induced rotational activity was recorded for 60 min following an injection of 2.5 mg/kg (+) methamphetamine HCl. The number of clockwise and counterclockwise turns were counted and expressed as the number of net rotations to the lesioned (ipsilateral) hemisphere (net rotations to ipsilateral hemisphere  =  ipsilateral rotations – contralateral rotations). KO animals exhibited a significant reduction in ipsilateral rotational behavior when compared to control animals at 2 and 4 weeks after the lesion (Fig. 9).

## Discussion

Mice with targeted ablation of *Pten* in DA neurons provide a unique opportunity to study the correlation between deregulated PTEN signaling and specific changes in DA neurotransmission. While the most described function of PTEN is mediated by the inhibition of cell-survival promoting PI3K/Akt- dependent signals, recent studies have shown PTEN regulates diverse functions in DA neurons, and its relationship to Parkinson’s disease and drug addiction has been highlighted. In this study, we have further characterized PTEN function specific to postmitotic mouse mesencephalic DA neurons. We generated a knockin mouse expressing Cre recombinase under the transcriptional control of the endogenous DAT promoter, to mediate restricted DNA recombination events [15]. *Pten* was selectively deleted in DA neurons in parallel to activation of the dopamine transporter at embryonic day 15. In addition, to allow bicistronic mRNA expression encoding for both DAT and Cre, we targeted the 3′ untranslated region (3′UTR) of the DAT gene where the Cre gene is preceded by an internal ribosomal entry site (IRES). *Pten* KO mice showed an enlarged ventral mesencephalon, neuronal hypertophy and increased numbers of DA neurons. Despite the robust trophic effects accompanied by a significant increase in overall striatal DA tissue content, extracellular DA levels in the striatum remained unchanged between control and *Pten* KO mice, and potassium evoked DA release was not elevated in *Pten* KO animals. However, DRD1 and PDyn mRNA levels were significantly elevated in striatonigral projecting neurons. PTEN inactivation protected DA neurons during exposure to a neurotoxin. This initial study of PTEN deregulation selective to DA neurons is aimed at describing the state of dopaminergic transmission and molecular adaptations of the mesostriatal and mesolimbic dopaminergic systems. The effects of PTEN deletion in DA neurons, as well as postsynaptic regions will be discussed in detail below.

### Disruption of PTEN activity in DA neurons during postmitotic developmental growth

Cell death by apoptosis is a normal developmental event occurring in most neuronal populations, and it is a determinant of the eventual size of a neuronal population. Developmental cell death is often regulated by the trophic synaptic support that neurons receive from their postsynaptic targets [31], [32], as well as from afferent projections [33]. Pruning of DA neurons in the mouse ventral midbrain is essentially a postnatal event with the number of apoptotic neurons culminating within the first days of life [31]. A second peak of apoptotic death among dopaminergic neurons occurs at postnatal day 14, a developmental period corresponding to competition for synapse formation [34]. In addition, and consistent with the model of target-derived support, excitotoxic striatal lesions during development result in a reduced number of nigro-striatal DA neurons. This decrease occurred in spite of the axon-sparing nature of the lesion [35], [36]. Thus, several lines of evidence suggest trophic factors, synaptic interactions and apoptotic events may play a key role in shaping the anatomy of the ventral mesencephalon.

In this study, we have shown *Pten* deletion in differentiated DA neurons causes a significant increase in the number and size of surviving neurons in both the mesolimbic and nigrostriatal projecting pathways. A known function of PTEN is antagonizing cell survival signals mediated by Akt-dependent pathways. Akt, or protein kinase B, exerts its role in promoting cell survival and growth by targeting anti-apoptotic downstream signals [37], [38]. Because at the time of *Pten* deletion DA neurons have already completed mitosis and phenotypic determination, it is unlikely the reported increase in DA neurons is due to an increase in newly formed neurons. It is thus likely PTEN ablation preserves DA neurons that normally would undergo apoptosis due to the lack of target support, by repressing the initiation of apoptotic pathways.

### While *Pten* deletion does not alter DA release, it induces permanent changes in striatal postsynaptic markers

Ablation of PTEN in DA neurons did not only cause robust anatomical changes in the ventral mesencephalon, but also affected functional connections between DA neurons and target areas. Interestingly, PTEN ablation did not generate changes in TH optical density in the striatum or nucleus accumbens, suggesting an equal distribution of dopaminergic terminals between *Pten* KO and control animals. *In vivo* microdialysis studies showed no differences in basal extracellular DA levels or in the dynamics of neurotransmitter clearance in the dorsal striatum. In addition potassium evoked depolarizations did not show an increase in DA release in *Pten* KO animals when compared to controls.

While no changes in DA release or uptake could be detected by microdialysis, KO animals showed a significant increase in total DA tissue content in dorsal striatum, an effect accompanied by a significant increase in striatal prodynorphin and DRD1 mRNA expression levels. As previous studies have shown enhancement of DA neurotransmission often results in elevated dynorphin and DRD1 mRNA levels in direct striatal projecting neurons, the unbalance between the direct and indirect striatal projection pathways observed in *Pten* KO animals could be explained by subtle but long-term changes in DA dynamics. In addition, we cannot rule out the possibility that the molecular changes specifically observed in dynorphin positive medium spiny neurons may be due to the direct synaptic interaction with DA neurons in the ventral mesencephalon. Medium spiny neurons may depend on trophic support and the availability of synaptic connections with their target, the SN, to define their molecular profile or even to survive during development. As *Pten* deletion creates a very robust change in substantia nigra, it is possible that the nigra may be in a better position to support afferent populations, such as the medium spiny neurons. Increased BDNF mRNA levels in the ventral mesencephalon in *Pten* KO animals, suggests that striatal afferents may have increased availability to trophic support in KO animals.

As locomotor activity and exposure to a novel environment are closely associated with mesocortical and mesolimbic activity [39], [40], we compared the locomotor responses of control and *Pten* KO animals when exposed to a novel environment. Interestingly, and despite higher DA contents in the striatum, *Pten* KO animals did not behave differently than controls when exposed to a novel environment. Increased DRD1 and dynorphin levels in medium spiny neurons may enhance the inhibition of DA neurons in the SNc, thereby normalizing overall DA release in the striatum.

### Suppressing PTEN expression protects DA neurons against exposure to a neurotoxin

The relationship between PTEN and Parkinson’s disease has been emphasized in several studies often in reference to mitochondrial function. PTEN downregulation in cultured hippocampal neurons inhibits the release of cytochrome c during toxic insults [41]. PTEN directly regulates the activity of PTEN-induced putative kinase 1(PINK), a protein kinase that localizes to mitochondria where it inhibits cytochrome c release, and known to be mutated in certain forms of familial Parkinson’s disease [42]. In addition, as an upstream modulator of Akt, the phosphatase PTEN has been shown to play a role in the regulation of survival signaling during neuronal injury. A previous study overexpressed a constitutively active form of the oncoprotein Akt/PKB in neurons of the ventral midbrain of adult mice using adeno-associated virus transduction [12]. The authors reported a significant increase in cell size and number of TH positive profiles in the SNc on the injected side. In addition, TH immunostaining showed higher density in the striatum ipsilateral to the adenoviral injection. There was also near complete protection of DA neuron cell bodies after intrastriatal 6OHDA lesion [12]. Our study provides further evidence that ablating PTEN activity leads to decreased dopaminergic neuronal death after a neurotoxic lesion. In agreement with the above mention study, we showed that *Pten* deletion specific to DA neurons confers significant protection against 6OHDA mediated toxicity [43], [44]. PTEN less DA neurons displayed a significant increase in survival rate as compared to control DA neurons. In addition, striatal TH density levels were significantly elevated in *Pten* KO animals as compared to controls. This data correlates with behavioral parameters, as methamphetamine induced rotations were significantly decreased in KO animals, suggesting increased maintenance of DA release in the lesioned striatum.

The demonstration that PTEN/Akt mRNA expression is modulated in response to different apoptotic inducers [7] strongly suggests the association of this pathway with neuronal survival and death. However, in recent years investigations have shown cellular functions attributed to PTEN in various neuronal circuits go beyond interactions with the Akt pathway, and have strengthened the current view of this molecule as a potential therapeutic target. For example, it has been recently shown that PTEN can physically interact with serotonin receptors in dopamine neurons to modulate transmission [45], [46]. Such interactions were able to affect the response of DA neurons to drugs of abuse, and therefore manipulation of the PTEN pathway could potentially affect the manner in which humans respond to reward and motivation. In addition, PTEN activity has been related to mitochondrial function in different neuronal systems, suggesting PTEN deregulation may provide a dual neuroprotective function in dopamine cells by the activation of survival pathways and maintenance of mitochondrial function. This molecule may thus have activity not only on cell death/survival, but also on other brain activities. Further studies defining the function of PTEN in dopamine cells may provide the molecular basis for the development of novel therapeutic strategies to treat CNS diseases.
